# Hypoxic regulation of the noncoding genome and NEAT1

**DOI:** 10.1093/bfgp/elv050

**Published:** 2015-11-20

**Authors:** Hani Choudhry, David R. Mole

**Keywords:** hypoxia, HIF, noncoding RNA, cancer

## Abstract

Activation of hypoxia pathways is both associated with and contributes to an aggressive phenotype across multiple types of solid cancers. The regulation of gene transcription by hypoxia-inducible factor (HIF) is a key element in this response. HIF directly upregulates the expression of many hundreds of protein-coding genes, which act to both improve oxygen delivery and to reduce oxygen demand. However, it is now becoming apparent that many classes of noncoding RNAs are also regulated by hypoxia, with several (e.g. micro RNAs, long noncoding RNAs and antisense RNAs) under direct transcriptional regulation by HIF. These hypoxia-regulated, noncoding RNAs may act as effectors of the indirect response to HIF by acting on specific coding transcripts or by affecting generic RNA-processing pathways. In addition, noncoding RNAs may also act as modulators of the HIF pathway, either by integrating other physiological responses or, in the case of HIF-regulated, noncoding RNAs, by providing negative or positive feedback and feedforward loops that affect upstream or downstream components of the HIF cascade. These hypoxia-regulated, noncoding transcripts play important roles in the aggressive hypoxic phenotype observed in cancer.

## Introduction

Both excessive and insufficient levels of oxygen are detrimental to cell biology, and so cells have developed tightly coordinated homeostatic mechanisms to respond to altered oxygen concentration. Chief amongst these is the regulation of gene expression by the hypoxia-inducible factors (HIFs) [1]. HIFs are heterodimers containing a regulated HIF-α subunit (HIF-1α, HIF-2α or HIF-3α) and a constitutive β-subunit (HIF-1β also called aryl hydrocarbon nuclear translocator, ARNT) [2–4]. Of the three HIF-α subunits, the functions of HIF-1α and HIF-2α are best characterized. Indeed, HIF-1 and HIF-2 regulate distinct but overlapping transcriptional profiles [5–10] comprising many hundreds of protein-coding genes that act to restore oxygen levels by reducing oxygen demand and increasing oxygen delivery.

**Figure 1.**
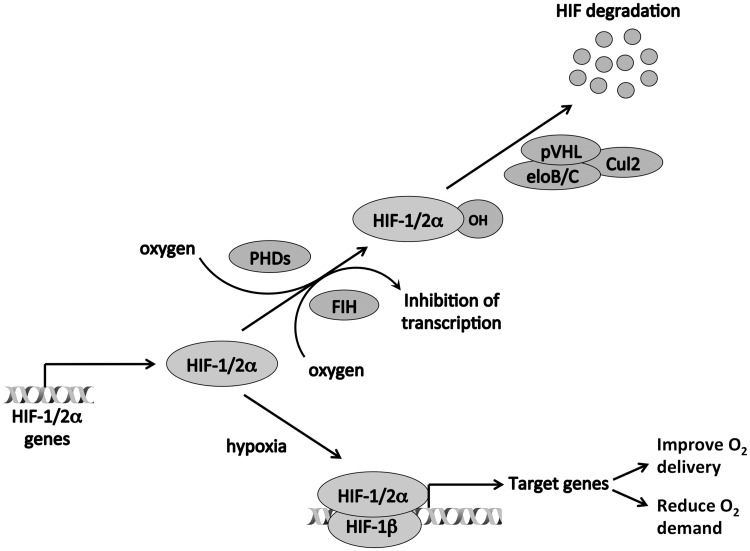
Regulation of transcription by HIF. In normoxia, HIF-α subunits are hydroxylated by both PHDs and by FIH. HIF-α that has been hydroxylated by the PHDs is recognized by pVHL, ubiquitinated and destroyed in the proteasome. Hydroxylation by FIH blocks the interaction between HIF-α and p300/CBP, inhibiting the transcriptional activity of HIF. In hypoxia, hydroxylation of HIF-α subunits is impaired, leading to their accumulation, dimerization with HIF-1β, binding to HREs and transactivation of target genes.

Lack of oxygen or tissue hypoxia is a key feature of many of the major causes of morbidity and mortality in the developed world, including myocardial and cerebral ischemia, and cancer [11, 12]. In atheromatous disease, oxygen delivery is reduced as a consequence of poor blood supply, while in solid tumors, the unregulated growth of malignant cells both increases oxygen demand and impairs perfusion by increasing diffusion distance from blood vessels to the cells, leading to upregulation of HIF [13–15]. There are multiple lines of evidence linking tumor hypoxia and the consequent HIF activation with an aggressive phenotype in cancer [16]. Firstly, tumor hypoxia and HIF levels are both associated with poor prognosis as well as resistance to chemotherapy and radiotherapy across many types of cancer [16]. Secondly, the transcriptional targets of HIF include genes with key roles in oncogenic processes such as angiogenesis, immortalization and self-renewal, epithelial to mesenchymal transition, metabolic reprogramming and invasion and metastasis [17]. Thirdly, in a wide range of tumor types, HIF loss-of-function generally results in decreased tumor xenograft growth, while HIF gain-of-function has the opposite effect, implying a causative role for HIF [16]. However, this association is not absolute, and in some tumor types, HIF-1 and HIF-2 may have opposing effects on xenograft growth [8, 18], while autochthonous mouse models also show mixed consequences of HIF inactivation on tumor behavior [19, 20]. Finally, many oncogenic and tumor-suppressor pathways directly affect HIF levels. The most direct and profound of these is the von Hippel Lindau (VHL) tumor suppressor, which is inactivated in the majority of clear cell renal cancers [21, 22]. This protein is the recognition component of a ubiquitin E3 ligase that targets HIF-α subunits for rapid degradation [23–27]. In the presence of oxygen, HIF-α subunits are posttranslationally modified by a family of prolyl hydroxylase domain-containing (PHD1-3, also known as Egl-9 family hypoxia inducible factor 1-3, EGLN1-3) 2-oxoglutarate-dependent dioxygenase enzymes [28–30] (Figure 1). pVHL specifically recognizes these hydroxylated HIF-α subunits but not the unmodified proteins that prevail in hypoxia [31]. Inactivation of VHL leads to high levels of HIF-α that mimic the hypoxic response and contribute to the pathogenesis of this disease [23]. In addition, another 2-oxoglutarate-dependent dioxygenase, factor inhibiting HIF (FIH), hydroxylates the C-terminal transactivation domain of HIF [32–34], blocking its interaction with p300/CBP [35–37]. However, HIF-1α and HIF-2α have a second N-terminal transactivation domain that is not targeted by FIH [38, 39], and therefore FIH has variable effects on hypoxic gene activation [40–42]. In hypoxia, hydroxylation of HIF-α subunits is impaired, leading to their accumulation, dimerization with HIF-1β, binding to hypoxia response elements (HREs) and transactivation of target genes [43].

## The HIF transcriptional output

Given the importance of HIF pathways to the pathogenesis of cancer, it is not surprising that considerable effort has gone into defining the transcriptional output of these factors. To date, most work has focused on hypoxic regulation of protein-coding genes (largely outside the scope of this article and well reviewed elsewhere). This approach initially concentrated on candidate genes [43] but subsequently used pangenomic microarray analyses [9] and more latterly high-throughput RNA-sequencing (RNA-seq) [44]. In recent years, this has been coupled with pangenomic analyses of HIF DNA-binding using chromatin immunoprecipitation (ChIP) coupled to tiled microarrays (ChIP-chip) or high-throughput sequencing (ChIP-seq) in attempts to distinguish direct from indirect transcriptional regulation [44–49]. This has revealed a number of previously unrecognized features of the HIF transcriptional landscape. Firstly, HIF binding may occur a long way from any annotated transcriptional start site. This binding is still associated with gene regulation, even over genomic distances in excess of 100 kb. Despite this, many regulated genes do not have identifiable HIF binding in their vicinity, indicating indirect mechanisms of regulation. Indeed, HIF binding is associated with upregulation, but not downregulation, of genes, indicating that gene suppression by HIF is largely indirect. Furthermore, within the confines of microarray technology or polyA-selected RNA sequencing that largely focused on the coding transcriptome, there were many ‘orphan' HIF-binding sites with no significantly regulated coding genes in the vicinity. This together with an increasing recognition of the importance of the noncoding transcriptome has raised the possibility that HIF DNA-binding might be regulating noncoding transcripts that could in turn be contributing to indirect regulation of the coding transcriptome.

With completion of the Human Genome project, it was recognized that only about 1.1% of the human genome encodes for RNAs that produce proteins. While much of the remaining genome may be structural and/or regulatory, recent advances in high-throughput sequencing technologies coupled to RNA analysis have identified increasing numbers of noncoding RNAs (ncRNAs) that do not code for proteins [50]. These include well-recognized classes of RNA such as ribosomal RNA (rRNA) and transfer RNA (tRNA) as well as new classes of small RNAs, including micro RNA (miRNA), piwi-interacting RNA (piRNA), small nucleolar RNA (snoRNA), enhancer RNA (eRNA) and a loosely defined group of long (>200 bp) noncoding RNAs (lncRNA) that also includes antisense RNA (asRNA) transcripts.

With the exception of miRNA expression, there has been little pangenomic analysis of the hypoxic response to systematically include noncoding classes of RNA because the majority will not be captured on microarrays or will be omitted by poly-adenosine and/or size selection during the preparation of sequencing libraries. In one notable exception, strand-specific analysis of ribosome-depleted RNA from MCF-7 breast cancer cells identified approximately 43 000 coding and noncoding transcripts in normoxia and hypoxia, across six major classes of RNA. This analysis revealed that all classes of transcript were regulated by hypoxia with major class-specific differences [44]. In particular, several classes of transcript (tRNAs, snoRNAs and piRNAs) showed overall downregulation, the former potentially contributing to the inhibition of protein synthesis observed in hypoxic cells [51]. Conversely, messenger RNAs (mRNAs), lncRNAs and miRNAs showed overall upregulation, the latter possibly as a result of hypoxia-induced posttranslational modification of argonaute 2 (Ago2) [52] or HIF transcriptional activation of argonaute 4 (Ago4) [53]. Within each class, a number of transcripts demonstrated strong up- or downregulation compared with the average fold-change.

Analysis of HIF DNA-binding in the same cells by ChIP-seq demonstrated that 20–30% of HIF-binding sites were closer to the promoter of a noncoding than a coding gene, with HIF-2α in particular binding close to noncoding gene loci. Correlation with hypoxic gene regulation revealed significant associations between HIF binding and upregulation, but not downregulation, for both coding and lncRNAs. A similar, weaker but nonsignificant association, was also seen for miRNAs, which were fewer in number [44]. These pangenomic analyses strongly implicate the HIF transcription factors in the direct transcriptional regulation of both lncRNAs and miRNAs, as well as the coding transcriptome.

## Hypoxic regulation of miRNAs

miRNAs are small, single-stranded, regulatory RNA molecules of approximately 22 nucleotides in length that were first discovered in 1993 [54]. More than 2000 miRNAs have now been discovered in humans, and it is estimated that up to 30% of coding transcripts are regulated by miRNAs [55]. They are transcribed as longer, immature transcripts that undergo several stages of processing to form single-stranded RNA–protein complexes, each capable of regulating the stability or translation of multiple (often hundreds) coding transcripts [56].

A number of studies (summarized in Table 1) have examined the hypoxic regulation of miRNAs using microarrays [52, 57–64], quantitative polymerase chain reaction (qPCR) [65] and, more recently, high-throughput sequencing [44, 53, 66]. Although miR-210 stands out as being consistently and robustly induced by hypoxia across all studies, the overlap between regulated miRNAs from each study is low. This degree of overlap is comparable with that seen for the hypoxic regulation of coding RNAs and likely reflects a high degree of cell-type specificity in the transcriptional response to hypoxia and HIF across all classes of RNA. Indeed, consistent with this, pangenomic patterns of HIF binding also show similar cell-type-specific patterns. Within a given cell-type, the association between pangenomic patterns of HIF binding and miRNA upregulation strongly implicates transcriptional regulation by HIF in the hypoxic induction of miRNAs.

**Table 1.** Hypoxia-regulated miRNAs

Study	miRNAs upregulated by hypoxia	miRNAs downregulated by hypoxia
Hua (2006), nasopharyngeal carcinoma cells, DFOM treatment, 20 h	miR-15, miR-188, miR-210, miR-30d, miR-155, miR-181b	Let-7-e, Let-7-g, miR-16, miR-26b, miR-30b, Let-7-f, Let-7-a, Let-7-c, Let-7-d, miR-15b, miR-20a, miR-20b, miR-224
Kulshreshtha (2007), colon and breast cancer cells, 0.2% O2, 8–48 h	miR-103, miR-106a, miR-107, miR-125b, miR-181a, miR-181c, miR-192, miR-21, miR-210, miR-213, miR-23a, miR-23b, miR-24-1, miR-26a, miR-27a, miR-93, miR-181b, miR-195, miR-26b, miR-30b	
Hebert (2007), head and neck squamous carcinoma cells, 1% O2, 1 h or 5% O2, 8 h	Let-7-i, miR-148a, miR-148b, miR-15a, miR-191, miR-200a, miR-210, miR-214, miR-373, miR-429, miR-498, miR-563, miR-572, miR-628, miR-637, miR-7, miR-98, Let-7-e, Let-7-g, miR-30b	miR-195, miR-29b, miR-30e-5p, miR-374, miR-422b, miR-101, miR-122a, miR-141, miR-186, miR-197, miR-19a, miR-320, miR-424, miR-565
Donker (2007), primary human cytotrophoblasts, 1% O2, 48 h	miR-125a, miR-152, miR-188, miR-191, miR-193b, miR-200b, miR-206, miR-210, miR-213, miR-23a, miR-23b, miR-27b, miR-30a-5p, miR-30c, miR-30d, miR-339, miR-452, miR-491, miR-512-5p, miR-93	miR-150, miR-155, miR-181b, miR-373, miR-128b, miR-181d, miR-196a, miR-196b, miR-200a, miR-25, miR-424, miR-449, miR-519e, miR-92, miR-489
Guimbellot (2009), colon cells, liquid–liquid interface.	let-7b, let-7e, miR-125a, miR-128a, miR-137, miR-148a, miR-185, miR-199a, miR-20, miR-204, miR-210, miR-213, miR-214, miR-23b, miR-26a, miR-299, miR-30a-3p, miR-30c, miR-335, miR-342, miR-150, miR-155, miR-16, miR-181b, miR-26b, miR-30b	miR-216, miR-9
Voellenkle (2012), HUVEC, 1% O2,	miR-210	
Choudhry (2014), breast cancer cells, 1% O2, 24 h	let-7b, let-7e, miR-103, miR-107, miR-151, miR-191, miR-193b, miR-210, miR-24-1, miR-27a, miR-27b, miR-30d, miR-339, miR-98, miR-181d	miR-125a, miR-15a, miR-200b, miR-342, miR-141
Camps (2014), breast cancer cells, 1% O2, 16, 32 and 48 h	miR-1, miR-106b-3p, miR-1246, miR-1269a, miR-140-3p, miR-141-5p, miR-143-3p, miR-151a-3p, miR-181c-3p, miR-192-5p, miR-194-5p, miR-195-3p, miR-203a, miR-215-5p, miR-27a-5p, miR-28-3p, miR-3065-3p, miR-30d-5p, miR-30d-3p, miR-30e-3p, miR-3140-3p, miR-3158-3p, miR-338-5p, miR-33b-5p, miR-203b-3p, miR-3619-3p, miR-3677-3p, miR-378c, miR-378d, miR-378i, miR-3913-5p, miR-3928-3p, miR-4504, miR-4746-5p, miR-4760-5p, miR-548a-3p, miR-627-5p, miR-92b-3p, miR-942-5p, miR-99b-5p, miR-24-2-5p, miR-27a-3p, miR-30b-3p, miR-30b-5p	miR-145-3p, miR-222-5p, miR-4521, miR-29b-1-5p, hsa-let-7f-1-3p, miR-1260a, miR-1260b, miR-1275, miR-15b-3p, miR-19a-3p, miR-19b-3p, miR-19b-1-5p, miR-22-3p, miR-221-5p, miR-23a-5p, miR-23b-5p, miR-296-3p, miR-32-3p, miR-33a-3p, miR-3613-5p, miR-424-3p, miR-4466, miR-455-3p, miR-505-5p, miR-573, miR-92a-1-5p, miR-93-3p, miR-940

Given the complex pleiotropy in miRNA responses (each miRNA can have multiple targets, which can be difficult to predict bioinformatically, and a specific coding transcript may be targeted by multiple miRNAs), it is not surprising that hypoxic regulation of miRNAs can have far-reaching effects on cancer cell biology. These are well reviewed elsewhere [67–69] and will therefore only be discussed briefly here.

Not surprisingly, miR-210, being the most ubiquitously hypoxia-induced miRNA, is also the best studied. It plays diverse roles in mediating HIF-regulated cell-cycle progression by targeting cell-cycle regulators, including E2F3 [70], MNT [71], FGFRL1 [72], PLK1, CDC25B, Cyclin F, BUB1B and FAM83D [73]. It also promotes hypoxia-induced angiogenesis through effects on ephrin-A3 (EFNA3) [74] and protein-tyrosine phosphatase 1b (PTP1B) [75]. Another important role is in the regulation of ISCU, which acts as a scaffold for the production of iron–sulfur clusters that are critical cofactors for enzymes involved in electron transport, the Krebs cycle and iron metabolism [76]. In addition, miR-210 may directly target components of the electron transport chain, such as NADH dehydrogenase (ubiquinone) 1 alpha subcomplex 4, succinate dehydrogenase complex, subunit D and cytochrome c oxidase assembly homolog 10 [69]. Genetic instability is a hallmark of cancer, and hypoxia-inducible miRNAs help modulate DNA repair. For example, miR-210 can suppress levels of RAD52, which is a key factor in homology-dependent repair [77]. miR-210 also inhibits apoptosis, another hallmark of cancer and is thought to affect a number of proteins in this pathway [69], but most specifically, Casp8ap2 [78].

However, miR-210 is not the only hypoxia-inducible miRNA to affect cancer pathways. miR-373 is also HIF-inducible miRNA and leads to a reduction in the nucleotide excision repair protein, RAD23B [77] and may synergize with miR-210 to increase DNA damage and genetic instability. In addition, other HIF-inducible miRNAs such as miR-107, which targets Pdcd10 [78], can also regulate apoptotic pathways. Furthermore, miR-21 has been variably reported as a hypoxia-inducible miRNA and also has a pro-survival role [67]. miR-181b is induced by hypoxia in retinoblastoma cells and stimulates proliferation [64]. Hypoxic downregulation of miR-34a targets the Notch signaling pathway to promote epithelial-to-mesenchymal transition (EMT) [79]. miRNAs may also target the HIF pathway itself, and this is discussed.

## Hypoxic regulation of other small RNAs (piRNAs, snoRNAs)

Increasing numbers of classes of noncoding RNA have been described (e.g. promoter-associated small RNAs; TSS-associated RNAs, promoter upstream transcripts, transcription initiation RNAs, piRNA and snoRNA) [80]. These are largely of unknown function and have been little studied in hypoxia. Nevertheless, one pangenomic study has examined regulation of piRNAs and snoRNAs in hypoxia [44].

piRNAs are a large family of small, single-stranded, noncoding, regulatory RNAs that are found throughout the animal kingdom. They play a role in the inhibition of transposon mobilization, and their expression correlates with a poor outcome in cancer [81]. snoRNAs are intermediate-sized noncoding components of ribonucleoproteins that help target posttranscriptional modifications to specific rRNAs and also have emerging roles in cancer [80, 82]. Both piRNAs and snoRNAs exhibit overall downregulation in hypoxia. However, within each class, there is heterogeneous behavior with some transcripts failing to downregulate or even increasing their expression in hypoxia [44]. Despite this, no association was seen between these transcripts and HIF binding to suggest direct transcriptional regulation of these classes of RNA by HIF.

## Long noncoding RNAs

lncRNAs are a heterogeneous class of regulatory RNAs that are arbitrarily (because of RNA-seq library protocols that frequently exclude small RNAs) >200 bp in length. Several overlapping classes may be distinguished, including transcripts antisense to protein-coding genes (asRNA), transcripts associated with enhancers (eRNA), bidirectional promoter-associated transcripts and other long intergenic noncoding RNAs (lincRNAs). They may act in *cis* to regulate expression of neighboring genes (e.g. Xist) or in *trans* through both transcriptional and posttranscriptional mechanisms [e.g. HOX transcript antisense intergenic (HOTAIR)].

lncRNA expression is highly cell-type specific, and many are frequently aberrantly expressed in cancer [83]. A number of lncRNAs have oncogenic properties, and their overexpression promotes tumor development, progression and metastasis, while others may act as tumor suppressors and are down regulated in cancer [84]. Indeed, lncRNAs regulate a number of biological and physiological processes that drive tumor development. For example, HOTAIR lncRNA regulates tumor invasion and metastasis [85], SOX2-overlapping transcript (SOX2-OT) and focally amplified long noncoding RNA in epithelial cancer (FALEC or FAL1) lncRNAs are involved in maintaining cancer cell stemness [86, 87], and imprinted maternally expressed transcript (H19), steroid receptor RNA activator (SRA) and growth arrest-specific 5 (GAS5) regulate cell proliferation and apoptosis [88–90]. Furthermore, lncRNA expression is associated with both clinicopathological features and prognosis in a range of cancers [91]. Thus, abundant lncRNAs such as H19, Urothelial Carcinoma Associated 1 (UCA1), HOTAIR, MALAT1, and HIF1A-antisense transcript (HIF1A-AS) are attractive as potential biomarkers and/or therapeutic targets in cancer.

## Hypoxic regulation of lincRNAs

Despite this, comparatively little is known about the pangenomic hypoxic regulation of lncRNAs because standard protocols for library preparation omit nonpolyadenylated RNA and do not preserve information about transcriptional direction. Nevertheless, a number of largely oncogenic lincRNAs have been individually reported to be regulated by hypoxia (see Table 2).

**Table 2.** Select hypoxia-regulated lncRNAs

Study	lncRNA	Regulation	HIF dependent	Function
Yang (2013)	lncRNA-LET	Down	No—deacetylation of promoter	Downregulation leads to stabilization of nuclear factor 90 protein and cancer cell invasion
Thrash-Bingham (1999), Bertozzi (2011), Choudhry (2014), Chen (2015)	HIF1A-AS	Up	Yes—direct	Downregulates HIF1A mRNA
Matouk (2007, 2010)	H19	Up	Yes	EMT, cell migration and angiogenesis
Ferdin (2013)	HINCUTS	Up	Yes—direct	Promotes hypoxic cell proliferation
Yang (2014)	lncRNA-p21	Up	Yes	Promotes hypoxic glycolysis
Wang (2014)	lncRNA-AK058003	Up		Regulates SNCG in *cis* by demethylating its promoter and promotes hypoxia-induced metastasis
Xue (2014)	lncRNA-UCA1	Up	Yes—direct	Induces cell proliferation, migration and invasion and reduces apoptosis
Takahashi (2014)	linc-RoR	Up	Not known	Promotes HIF1A mRNA expression
Choudhry (2014), Michalik (2014)	MALAT1	Up	Yes—direct	Affects splicing patterns of alternative exons and promotes cellular proliferation, tumor growth, angiogenesis and metastasis
Gomez-Maldonado (2015)	lncRNA-EFNA3	Up	Yes—direct	Downregulates EFNA3, possibly by competing for miR-210
Zhou (2015)	HOTAIR	Up	Yes—direct	Enhances hypoxic cancer cell proliferation, migration and invasion
Choudhry (2014, 2015)	NEAT1	Up	Yes—direct	Induces nuclear paraspeckle formation, leading to cancer cell survival

H19 is an oncogenic lncRNA that is highly expressed in many cancers and has roles in EMT, cell migration and angiogenesis. H19 is induced by hypoxia through activation of HIF-1α in cooperation with wild type p53 [92, 93]. lncRNA-p21 is also induced in hypoxia again through transcriptional regulation by HIF-1α and modulates the Warburg effect by promoting hypoxic glycolysis [94]. lncRNA-AK058003, which lies 8.6 kb upstream of Synuclein gamma (breast cancer-specific protein)—SNCG—is also induced by hypoxia [95]. lncRNA-AK058003 regulates SNCG in *cis* by demethylating its promoter and promotes hypoxia-induced metastasis.

While these lncRNAs could be induced either directly or indirectly by HIF (or indeed in some cases by non-HIF-mediated mechanisms), there is good evidence that a number of them are direct transcriptional targets of HIF. For example, the lncRNA–Urothelial Carcinoma Associated 1 (lncRNA-UCA1) is induced by hypoxia through HIF-1α and induces cell proliferation, migration and invasion and reduces apoptosis [96]. Both electrophoretic mobility shift assays and ChIP have confirmed direct binding of HIF-1α to the lncRNA-UCA1 promoter. Using microarray analysis, Ferdin *et al*. identified five transcribed-ultraconserved regions that were induced by hypoxia and HIF, which they termed ‘hypoxia-induced noncoding ultraconserved transcripts' (HINCUTS) [97]. These highly conserved RNAs are upregulated in colon cancer and can promote hypoxic cell proliferation. Again, direct transcriptional activation by HIF has been confirmed by ChIPing the transcription factor at the promoters of several of these HINCUTs. Furthermore, using similar methodologies, HOTAIR itself has also been shown to be a direct transcriptional target of HIF, contributing to cancer cell proliferation, migration and invasion in hypoxia [98].

Some of these HIF-dependent lncRNAs may regulate protein-coding genes via complex mechanisms. For example, the EFNA3 gene also binds HIF directly, where in addition to the canonical protein-coding mRNA, two additional lncRNA transcripts are expressed from alternate promoters [99]. Rather than regulating the protein-coding gene directly, HIF transactivates the two lncRNAs, which increase EFNA3 protein levels and promote metastatic dissemination without affecting EFNA3 mRNA expression. Interestingly, translation of EFNA3 mRNA to protein is inhibited by the hypoxia-inducible miR-210 [74]. It is thought that the hypoxic induction of EFNA3 lncRNAs, which also contain the miR-210-binding site, dominantly competes miR-210 away from EFNA3 mRNA to release this repression. However, whether EFNA3-lncRNA levels are sufficient to do this and why such a complex mechanism might exist remain unclear.

In addition to hypoxic induction, lncRNAs can also be downregulated by hypoxia. Importantly, this appears to occur through indirect mechanisms rather than through direct transcriptional inhibition by HIF. For example, the lncRNA Low Expression in Tumors (lncRNA-LET) is downregulated in hypoxia as a consequence of hypoxic induction of histone deacetylase 3, leading to reduced acetylation of the lncRNA-LET promoter [100]. Low expression of lncRNA-LET is a common feature of hepatocellular, colorectal and squamous cell lung carcinomas and is a key step in the stabilization of nuclear factor 90, which leads to hypoxia-induced cancer cell invasion.

Increasingly systematic approaches have identified growing numbers of hypoxia-regulated lncRNAs. Furthermore, given the cell-type specificity of lncRNA expression and of the regulation of other classes of RNA, it is likely that there are significantly more to be discovered. Takahashi *et al*. examined the hypoxic regulation of 89 lncRNAs by qPCR, of which 20 were significantly upregulated and 18 downregulated [101]. However, they did not investigate the mechanisms by which these lncRNAs were regulated. Wang *et al*. analyzed the pangenomic lncRNA response to hypoxia by microarray and identified 84 lncRNAs that were upregulated and 70 that were downregulated >1.5-fold when compared with normoxic cells [95]. More recently, Choudhry *et al*. undertook a combined RNA-seq and ChIP-seq analysis in normoxic and hypoxic MCF-7 breast cancer cells to determine both the extent of hypoxic regulation of ncRNAs and the involvement of the HIF transcription factors in this regulation [44]. HIF binding was associated with hypoxia-induced lncRNAs, but not hypoxia-downregulated lncRNAs in a pattern reminiscent of coding transcripts. This confirms a major role for HIF in the direct transactivation, but not transrepression, of lncRNAs as well as protein-coding transcripts. Furthermore, HIF-2-binding sites were more likely than HIF-1-binding sites to be close to lncRNA promoters, suggesting that HIF-2 plays a greater role in the regulation of the noncoding transcriptome than HIF-1.

## Hypoxic regulation of metastasis-associated lung adenocarcinoma transcript 1 and nuclear enriched abundant transcript 1

In this and other studies, two of the most hypoxia-induced targets of the HIF transcription factor were metastasis-associated lung adenocarcinoma transcript 1 (MALAT1, also known as NEAT2) and nuclear enriched abundant transcript 1 (NEAT1) [44, 102, 103]. These are both members of a subgroup of highly conserved lncRNAs that stably and abundantly localize to distinct nuclear bodies [104] and are expressed from neighboring single exon genes on chromosome 11q with each having its own HIF-binding site.

MALAT1 localizes to nuclear structures known as nuclear speckles, although the formation of these structures is not dependent on its presence [105]. However, MALAT1 interacts with serine-/arginine-rich splicing factors (SRSF), including serine-/arginine-rich splicing factors-1, 2 and 3 (SRSF1, SRSF2 and SRSF3) [106], and is responsible for their recruitment to the nuclear speckles, where they affect the splicing patterns of alternative exons [104]. However, whether the hypoxic induction of MALAT1 contributes to alternate patterns of splicing in hypoxia is unknown. In addition, MALAT1 is also the precursor of a conserved cytoplasmic tRNA-like small RNA, MALAT1-associated small cytoplasmic RNA, of unknown function. MALAT1 is widely expressed and is frequently upregulated or mutated in solid tumors in which it promotes cellular proliferation, tumor growth and metastasis [107]. Furthermore, hypoxic upregulation of MALAT1 in endothelial cells contributes to the angiogenic response, indicating that MALAT1 may play an important role in tumor angiogenesis [108].

NEAT1 is an architectural component of nuclear paraspeckles, which lie adjacent to nuclear speckles. There are two NEAT1 transcripts, NEAT1_1 and NEAT1_2, also known as multiple endocrine neoplasia ε and β, which differ only in their 3’-end [109]. The shorter 3.7 kb form, NEAT1_1, is polyadenylated and widely expressed in different mammalian tissues [110]. NEAT1_2 is 23 kb long, and its 3′-tail is cleaved off by RNAse P to leave a triple helical remnant that is critical for its stability [111]. Because each form is expressed from the same promoter, they are both transcriptionally regulated by HIF in the same way [112]. Interestingly, although both HIF isoforms bind at the NEAT1 locus, NEAT1 is regulated predominantly by HIF-2 rather than HIF-1, indicating post-binding mechanisms of transcriptional selectivity.

Because NEAT1 (specifically NEAT1_2) is required for the formation of paraspeckles [111, 113–115], its HIF-2-dependent induction leads to the increased formation of paraspeckles in hypoxia [112]. The biological functions of paraspeckles are currently poorly understood, but they are thought to have regulatory roles in gene expression, by affecting both transcription and translation [116–119]. Paraspeckles are rich not only in NEAT1, but also in RNA-binding proteins, including RNA-binding motif protein 14, paraspeckle component 1, non-POU domain containing, octamer-binding protein (NONO or p54nrb) and splicing factor proline-/glutamine-rich protein [110], which are recruited to the paraspeckles by NEAT1 in hypoxia [112]. These RNA-binding paraspeckle proteins can bind transcripts that have been subjected to A-to-I editing within Alu repeat elements, retaining them in the nucleus and potentially inhibiting their translation [118, 119]. In addition, sequestration of other multifunctional protein components in paraspeckles can deplete their levels and inhibit their activity in the nucleoplasm [116, 117]. Notably, the hypoxic induction of nuclear paraspeckles by HIF led to the nuclear retention of F11R (junctional adhesion molecule 1, JAM1) transcripts [112, 120]. The extent to which hypoxic induction of nuclear paraspeckles contributes to the indirect regulation of other HIF-dependent genes that do not directly bind HIF remains to be determined. However, hypoxic induction of NEAT1 promotes cell proliferation and survival and inhibits apoptosis, while high expression of NEAT1 in breast cancer is associated with a poor prognosis [112].

## Hypoxic regulation of asRNAs

asRNAs are a subclass of lncRNAs that overlap with protein-coding genes, but are transcribed from the opposite strand. They can control nearly every level of gene regulation, including pretranscriptional, transcriptional and posttranscriptional, through DNA–RNA, RNA–RNA or protein–RNA interactions [121]. Several studies have described hypoxic upregulation of asRNAs through mechanisms that do not involve direct transcriptional activation by HIF. For example, Fish *et al*. demonstrated hypoxic induction of endothelial nitric oxide synthase (eNOS) asRNA (known as sONE, NOS3AS or APG9L2) through transcript stabilization [122] that in turn leads to suppression of eNOS expression. McCarthy *et al*. showed that demethylation of a CpG island in intron 1 of the Wilm’s tumor 1 gene in hypoxia leads to induction of an antisense lncRNA that is required for hypoxic induction of the protein-coding transcript [123].

However, until recently, a systematic analysis of the hypoxic regulation of antisense and sense transcripts pairs and the contribution of direct HIF-dependent transactivation had not been undertaken. Using strand-specific RNA-seq analysis coupled to ChIP-seq for the HIF transcription factors and RNApol2 has revealed numerous instances in which both the sense- and asRNA transcripts are regulated by hypoxia [44]. These may also be associated with direct binding of HIF, suggesting direct transcriptional regulation. The opposing protein-coding transcripts may be co-regulated in the same manner or counter-regulated with one transcript increasing in expression, while the other is reduced. However, the extent to which the asRNA transcripts contribute to the regulation of their partners remains largely undetermined.

## Regulation of the hif pathway by the noncoding transcriptome

**Figure 2.**
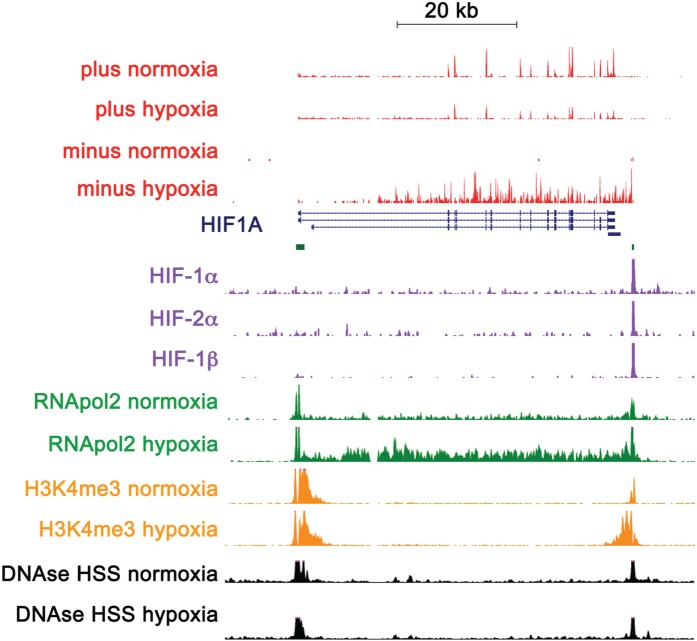
HIF-1A antisense transcript. Tracks for positive and negative strand RNA-seq in normoxia and hypoxia are shown in red. The positive strand HIF-1α mRNA is reduced in hypoxia, while the antisense HIF-1α is induced by hypoxia. Refseq genes are shown in navy with CpG islands identified at each end of the gene. HIF ChIP-seq tracks are shown in purple and show strong binding close to the TSS of the antisense transcript. RNApol2 ChIP-seq tracks, in green, show RNApol2 peaks at each end of the gene, with an increase in the right-hand peak together with increased RNApol2 across the body of the gene in hypoxia. H3K4me3 ChIP-seq tracks, in orange, show peaks of the promoter-associated mark at both ends of the HIF1A gene, with an increase in the right-hand peak in hypoxia. DNAse hypersensitivity tracks, in black, show peaks at both ends of the HIF1A gene. (A colour version of this figure is available online at: http://bfg.oxfordjournals.org)

One important example of a hypoxia-regulated asRNA is the anti-HIF-1α transcript [124–126]. The HIF-1α gene has a CpG island at both ends. Examination of DNA-accessibility (DNAse-seq) and ChIP-seq (H3K4me3 and RNApol2) indicates the presence of an active promoter at each end of the gene. In hypoxia, the spliced sense transcript is reduced. However, RNApol2 running across the gene is seen to increase along with the expression of an unspliced antisense transcript (Figure 2) [44]. The transcriptional start site of this antisense transcript is closely associated with a HIF-binding site, strongly suggesting that HIF-1α transcriptionally activates its own asRNA, which through undetermined mechanisms then downregulates expression of the sense transcript. In addition, Wang *et al*. [127] identified a promoter upstream transcript (TCONS_00004241) at the HIF-2α (EPAS1) locus that they termed HIF2A promoter upstream transcript (HIF2PUT). This positively correlated with and contributed to regulation of HIF-2α mRNA, indicating that both the major isoforms of HIF-α are regulated by noncoding RNAs. However, whether HIF2PUT is regulated by hypoxia/HIF leading to another feedback loop remains unknown.

**Figure 3.**
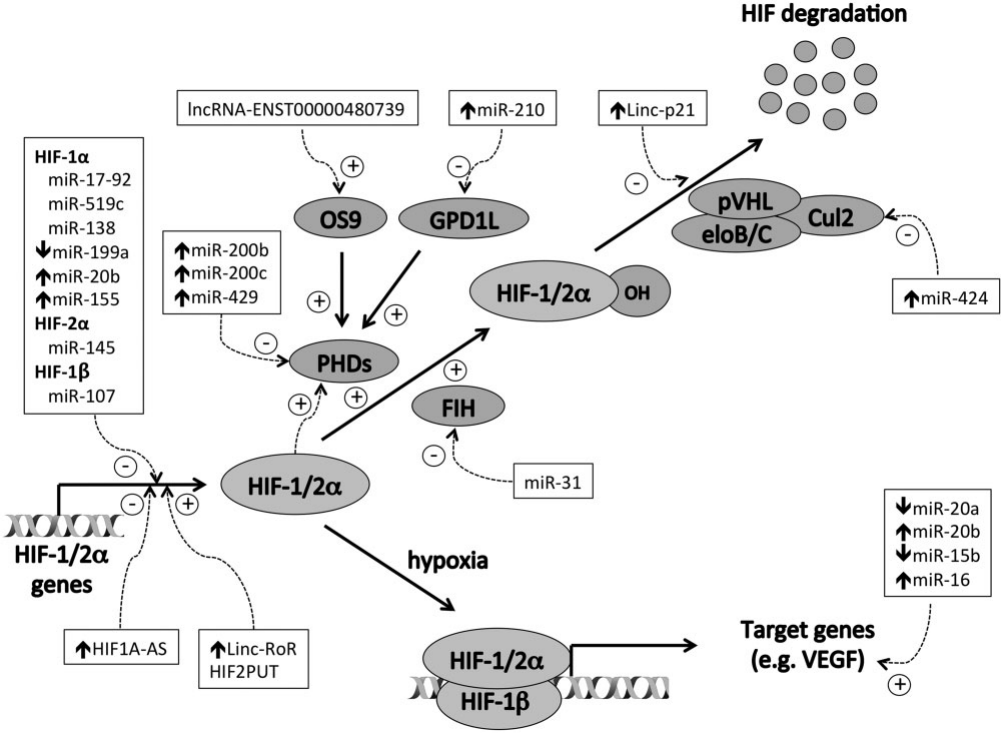
Regulation of the HIF pathway by noncoding RNAs. Protein components are shown in gray ovals. ncRNAs are shown in boxes, and their regulation by hypoxia is denoted by the short arrows ↑ and ↓ ncRNAs induce (+) and inhibit (−) multiple aspects of the HIF pathway.

Other lncRNAs also regulate the HIF transcription factors but in *trans* rather than in *cis* (Figure 3). For example, lncRNA-ENST00000480739 acts in *cis* to induce transcription of osteosarcoma amplified-9, which in turn acts in *trans* to suppress HIF-1α levels by increasing its degradation and thereby suppressing tumor cell invasion [128]. Conversely, linc-RoR, can promote HIF-1α mRNA expression and therefore augment the hypoxic transcriptional response [101]. Another hypoxia-inducible lncRNA, lincRNA-p21, is able to bind HIF-1α and pVHL and disrupt the HIF-1α–pVHL interaction, thereby augmenting HIF-1α protein levels by increasing protein stability [94]. lincRNA-p21 is itself a transcriptional target of HIF-1α generating a positive feedback loop that promotes HIF-dependent pathways such as glycolysis in hypoxia.

lncRNAs are not the only component of the noncoding genome that can feedback regulate the HIF pathway. miRNAs that downregulate HIF-1α include the miR-17-92 cluster [129], miR-138 [130], miR-199a [131, 132], miR-20b [133, 134], miR-519c [135] and miR-155 [63] and may contribute to the attenuation of HIF-1α activation in prolonged hypoxia. While these show specificity for HIF-1α, miR-145 selectively inhibits HIF-2α [67], potentially altering the balance between the two HIF isoforms in the opposing direction. In addition, miR-107 inhibits HIF-1β expression [136], thereby affecting the activity of both HIF-1 and HIF-2. Other miRNAs suppress negative regulators of HIF such as PHD2 (miR-200b, miR-200c, and miR-429) [137], FIH (miR-31) [138], Cul-2 (miR-424) [139] or GPD1L (miR-210) [140], leading to upregulation of the HIF transcriptional response.

In addition to generating feedback loops, miRNAs can also feedforward to modulate specific aspects of the HIF transcriptional response. For example, angiogenesis, which is central to the pathogenesis of cancer is orchestrated by vascular endothelial growth factor (VEGF), a direct transcriptional target of HIF. The hypoxia-regulated miRNAs miR-20a, miR-20b, miR-15b and miR-16 all target the 3’-end of VEGF mRNA and suppress translation [60, 137]. Hypoxic downregulation of these inhibitory miRNAs therefore coordinately augments the VEGF transcriptional response to hypoxia.

Thus, ncRNAs are not only effectors of the indirect response to HIF transcriptional activation, but also modulate the expression of direct transcriptional targets of HIF, in addition to the HIF transcription factors themselves. In addition to integrating inputs from other signaling pathways, this also creates complex positive and negative feedback loops that both augment and restrict the HIF response to hypoxia and are in addition to the negative feedback loops generated by the transcriptional activation of PHD2 and PHD3 by HIF. Because noncoding RNA expression is highly cell-type specific, it is likely that the number of ncRNAs influencing both HIF levels and HIF target genes will only increase with time.

## Conclusion

**Figure 4.**
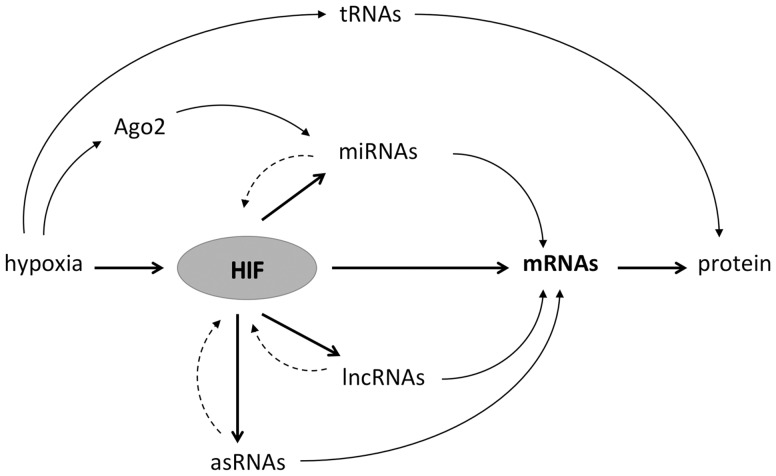
ncRNAs act as effectors and modulators of the HIF transcriptional pathway. In addition to directly transactivating mRNAs expression, HIF also induces the expression of miRNAs, lncRNAs and antisense sRNAs. These can in turn affect the expression of mRNAs or feedback on the HIF pathway itself. Hypoxia might also directly alter miRNA levels through hydroxylation of Ago2 or protein synthesis through the inhibition of tRNAs.

Noncoding transcripts and miRNAs and lncRNAs in particular are highly regulated by hypoxia and by HIF and, in turn, contribute to the regulation of the coding genome. Hypoxia-regulated ncRNAs may act on the coding genome either in *cis* or in *trans* and provide indirect routes to gene regulation by HIF (e.g. through chromatin modification, regulation of transcription or posttranscriptionally). Alternatively, they may act on direct transcriptional targets of HIF to augment their expression. In addition, ncRNAs may act on HIF itself or on its upstream regulators. This may integrate inputs from other regulatory pathways, or when these ncRNAs are themselves transcriptional targets of HIF, providing both positive and negative feedback loops that either augment or limit the HIF response or effect a switch in isoform expression (Figure 4).

Key Points

- Activation of hypoxia pathways orchestrated by the transcription factor HIF is associated with and contributes to an aggressive phenotype in many different cancers.
- In addition to protein-coding genes, HIF directly transactivates micro RNAs, long noncoding RNAs and antisense RNAs.
- These help regulate protein-coding transcripts, either by targeting specific mRNAs or by affecting generic RNA-processing pathways.
- Noncoding RNAs are both effectors of the hypoxia response and modulators of the HIF transcriptional cascade.
- Hypoxia-regulated, noncoding RNAs play an important role in the adverse hypoxic phenotype observed in cancer.

## Funding

This work was funded by the Deanship of Scientific Research (DSR), King Abdulaziz University, Ministry of High Education for Saudi Arabia, Cancer Research UK (A16016), the Higher Education Funding Council for England and the Ludwig Institute for Cancer Research.
